# The Impediment in Acquiring Clinical Skills by Medical Students during the COVID-19 Pandemic

**DOI:** 10.31729/jnma.5559

**Published:** 2021-01-31

**Authors:** Avilasha Singh

**Affiliations:** 1 Kathmandu Medical College and Teaching Hospital, Sinamangal, Kathmandu, Nepal

**Keywords:** *clinical competence*, *COVID-19*, *medical students*

## Abstract

With the country being in lockdown for almost seven months now, all the academic studies from school to college have been shifted online. While online learning has been going on fine with some adjustments and compromises, clinical education for medical undergraduates has been seriously affected. Without the proper student-patient interaction and hands-on-experiences, acquiring clinical skills by the students has been difficult.

With the country being in lockdown for almost 7 months now, all the academic studies from school to college have been shifted online. To students fortunate enough to have resources to attend classes, with slight adjustments and compromises, online learning has been going on somewhat fine. It has not drastically affected anyone's academic education. But infelicitously, the same does not apply for getting clinical skills knowledge by medical undergraduates. In a study done among undergraduates of a medical college in Nepal, 77.51% of the respondents rated that the online classes were not effective.^1^ In another similar study done among several Kathmandu University colleges, only 1% of the respondents strongly agreed that practical or clinical simulation exercise was possible through an online class, whereas 58.9% of the respondents strongly disagreed.^2^

Clinical postings are a huge part of the medical school curriculum. It is imperative to master clinical skills to become a competent practitioner. Intensive training in clinical skills right at the beginning of a medical career builds a good medical professional.^3^ This pandemic, however, has adversely affected it.

While theory teaching can be effectively conducted via an online medium, the same cannot be said for practical teaching. Practical teaching includes history taking, the examination of the patient, coming to a differential diagnosis after critical reasoning, maintaining good communication and professionalism. But due to the risk of crowding and transmission of infection, students have not been able to interact with the patients. A patient teaches a student better than a book. Social intelligence and confidence are things one cannot find in a book.^4^ Hence, not being able to see the patients in clinical rotations has been a big blow to the student's clinical knowledge. This has done a disservice to the students by preventing them from developing their inter-personal and clinical confidence.^4^

On top of that, there are several instruments that one needs to use while conducting a medical examination. For sure, procedures to use these instruments can be found in numerous tutorial videos all over the internet. The teachers can easily demonstrate them as well. But the most important part that got missed out is the hands-on-experience of the students. Without the students being able to hold the instrument themselves and use it, the demonstrations they observe become merely theoretical knowledge. It is very difficult for the students to put that knowledge into clinical practice. The experience of students holding and positioning the instruments, looking at the examination findings themselves is valuable. Any other form of learning cannot compensate for this.

Undergraduate knowledge is a foundation for future learning and practice in a medical career. To ensure a strong undergraduate foundation, it is paramount to focus on clinical and practical skills rather than acquiring factual knowledge.^5^ Since clinical postings are all about ‘learning by doing,’ the student's clinical education is compromised. It is important to find common ground to let medical undergraduates acquire the necessary clinical skill in a safe environment amidst the pandemic.
